# Supplementary material for: Quantifying the polygenic contribution to variable expressivity in eleven rare genetic disorders
Source: Nat Commun. 2019 Oct 25;10:4897. doi: 10.1038/s41467-019-12869-0 (PMC6814771; doi:10.1038/s41467-019-12869-0)
Supplement: Supplementary file 2 — Supplementary Information [file 41467_2019_12869_MOESM2_ESM.pdf]

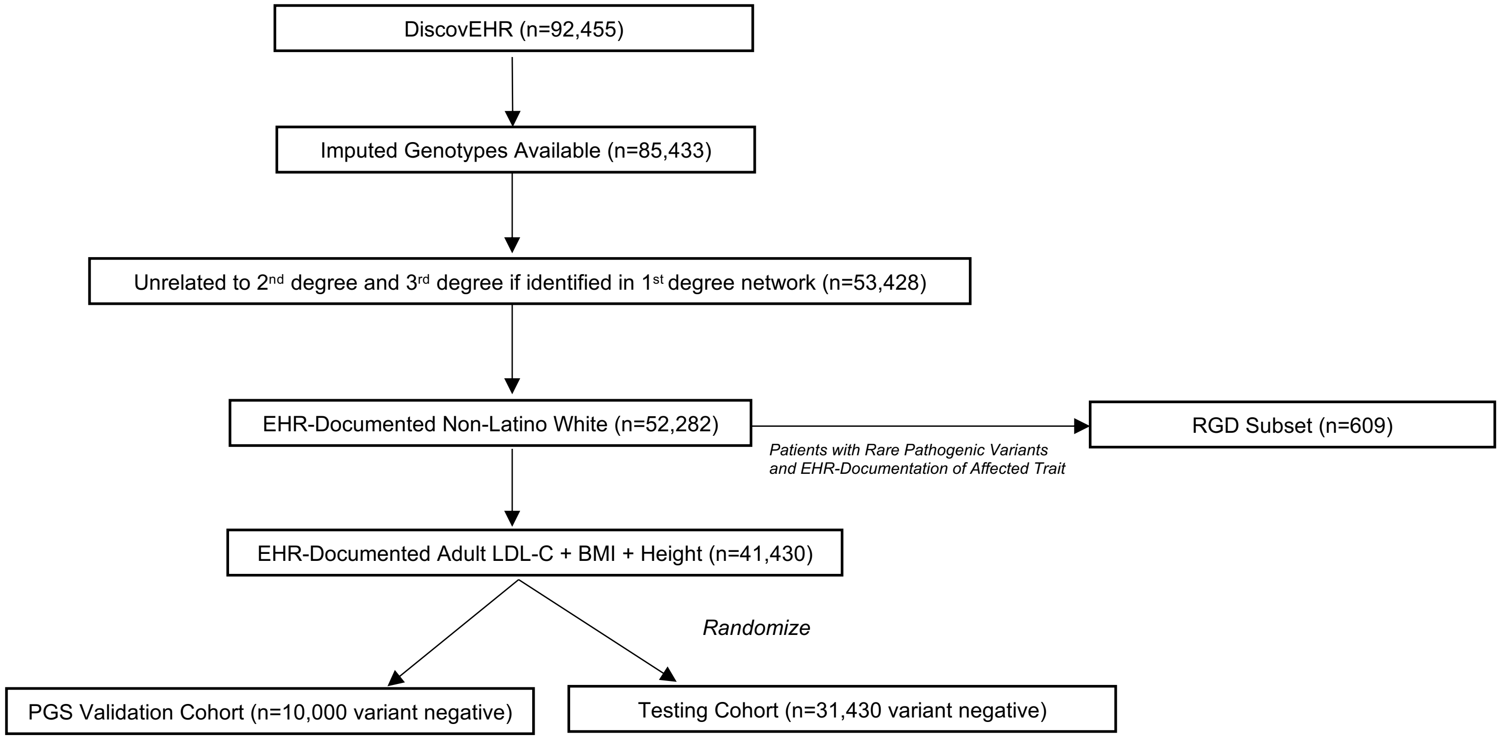

Supplementary Figure 1: Inclusion criteria for MyCode patient-participants in this study.

RGD: Rare Genetic Disorder
PGS: Polygenic Score

A.   B.

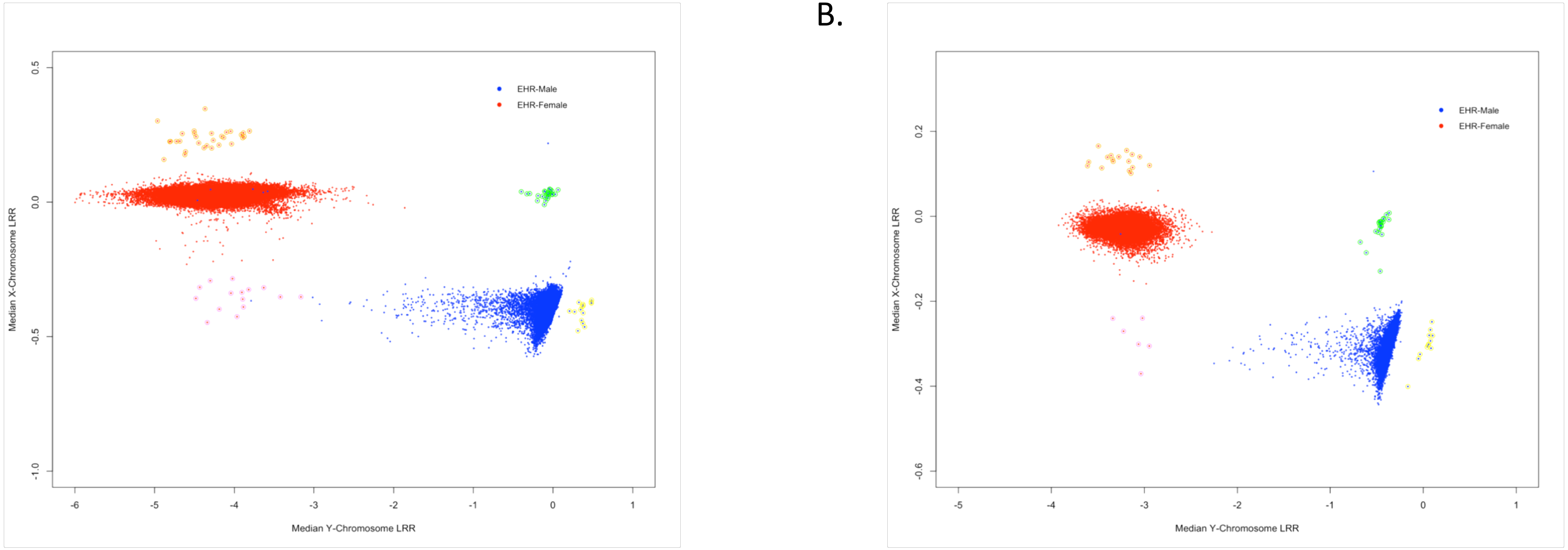

Supplementary Figure 2: Median X- and Y-chromosome Log R Ratios (LRR) of patient-participants genotyped on the Human Omni Express Exome (n=55,054) (A) and Global Screening Array (n=25,207) (B) passing QC. Points are colored based on EHR-documented gender for male (blue) and females (red). Sex chromosome aneuploidies are indicated with colored circles as followed: 47,XXX (orange), 47,XXY (green), 45,X + 45,X/45,XX (pink), and 47,XYY (yellow). The six EHR-documented males with median X- and Y-chromosome LRR values consistent with 46,XX were removed from further analysis.

A.

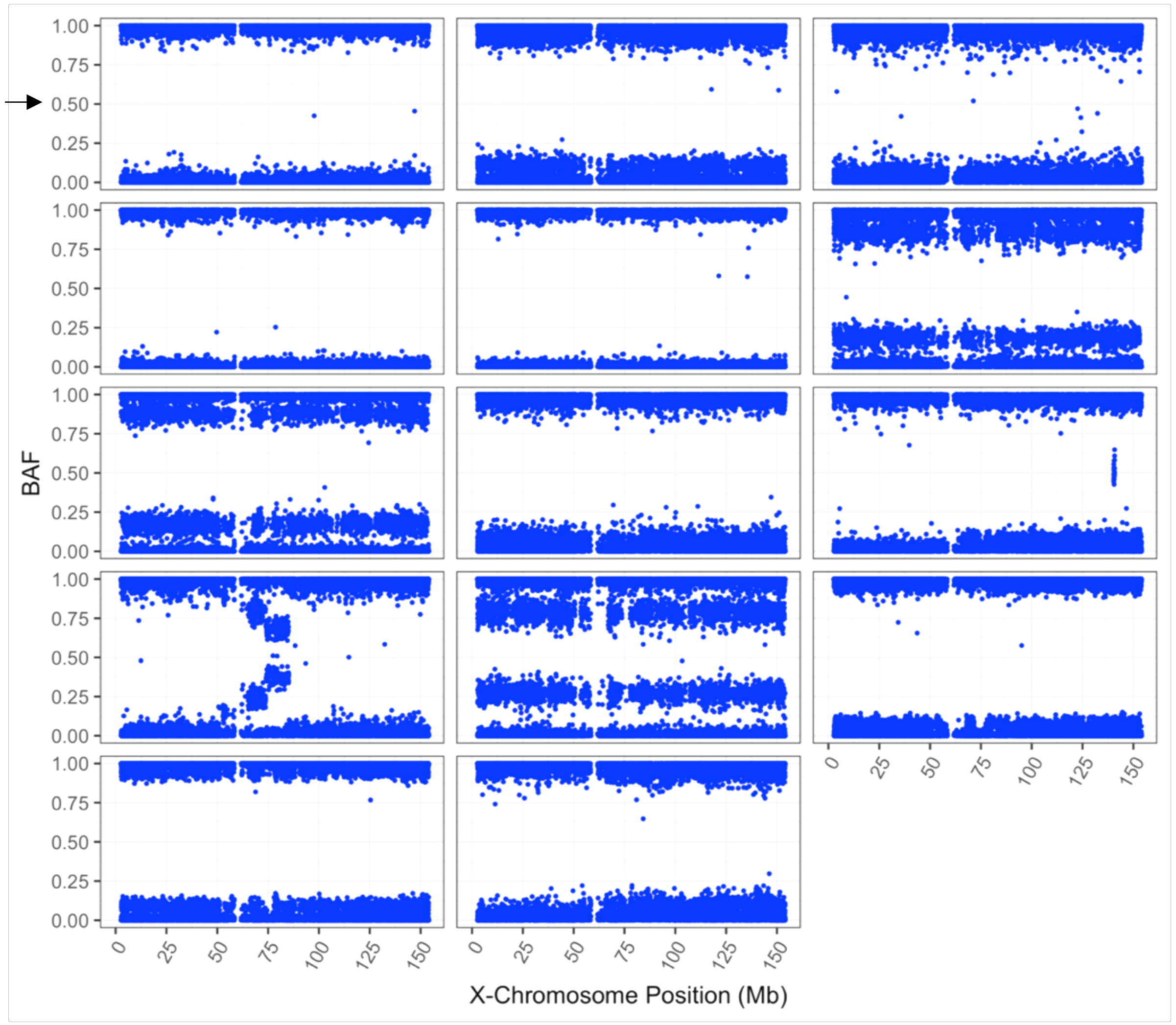

B.

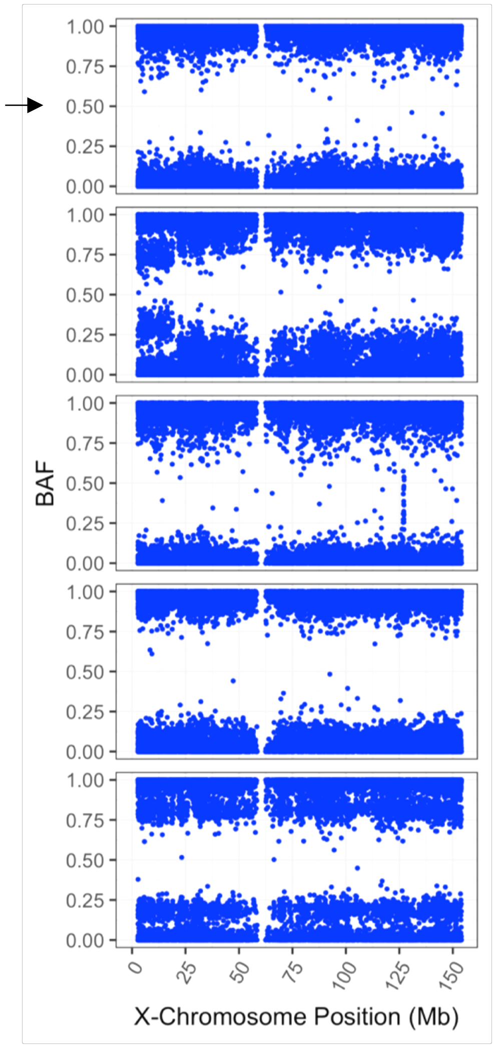

Supplementary Figure 3: X-chromosome B-allele frequency (BAF) profiles of the 45,X and 45,X/46,XX cases genotyped on the HOEE (A) and GSA (B) passing sample inclusion criteria and included in this study. Reference samples ($mLRR_{min}$) for each platform used for 100% loss to calculate mosaicism are indicated with a black arrow.

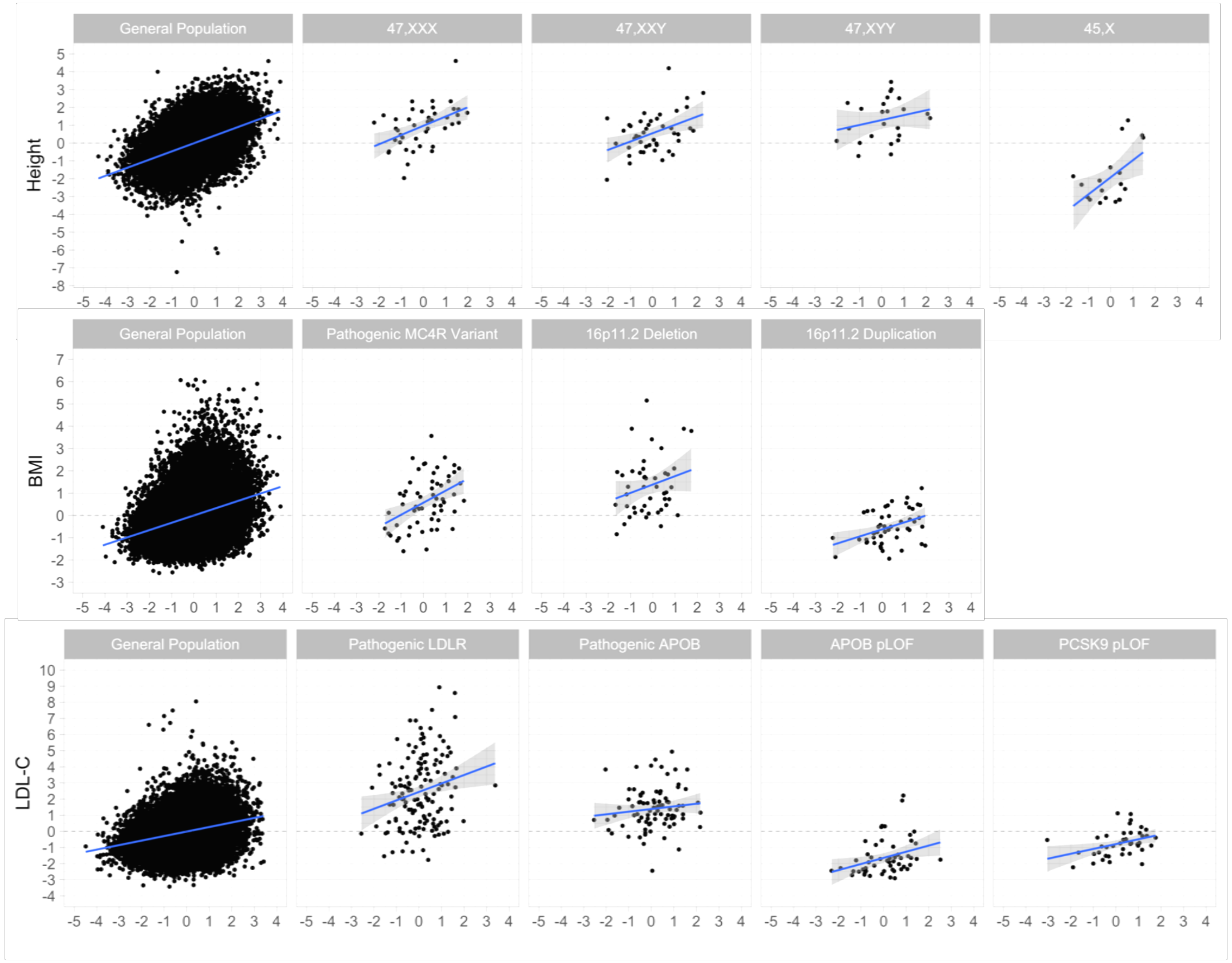

Supplementary Figure 4: Scatterplots of the standardized polygenic score (x-axis) against standardized quantitative phenotypes (y-axis). The regression line is indicated in blue and the gray shadow indicates the 95% confidence level interval. A horizonal dashed line is drawn in plots at 0 representing the population average.

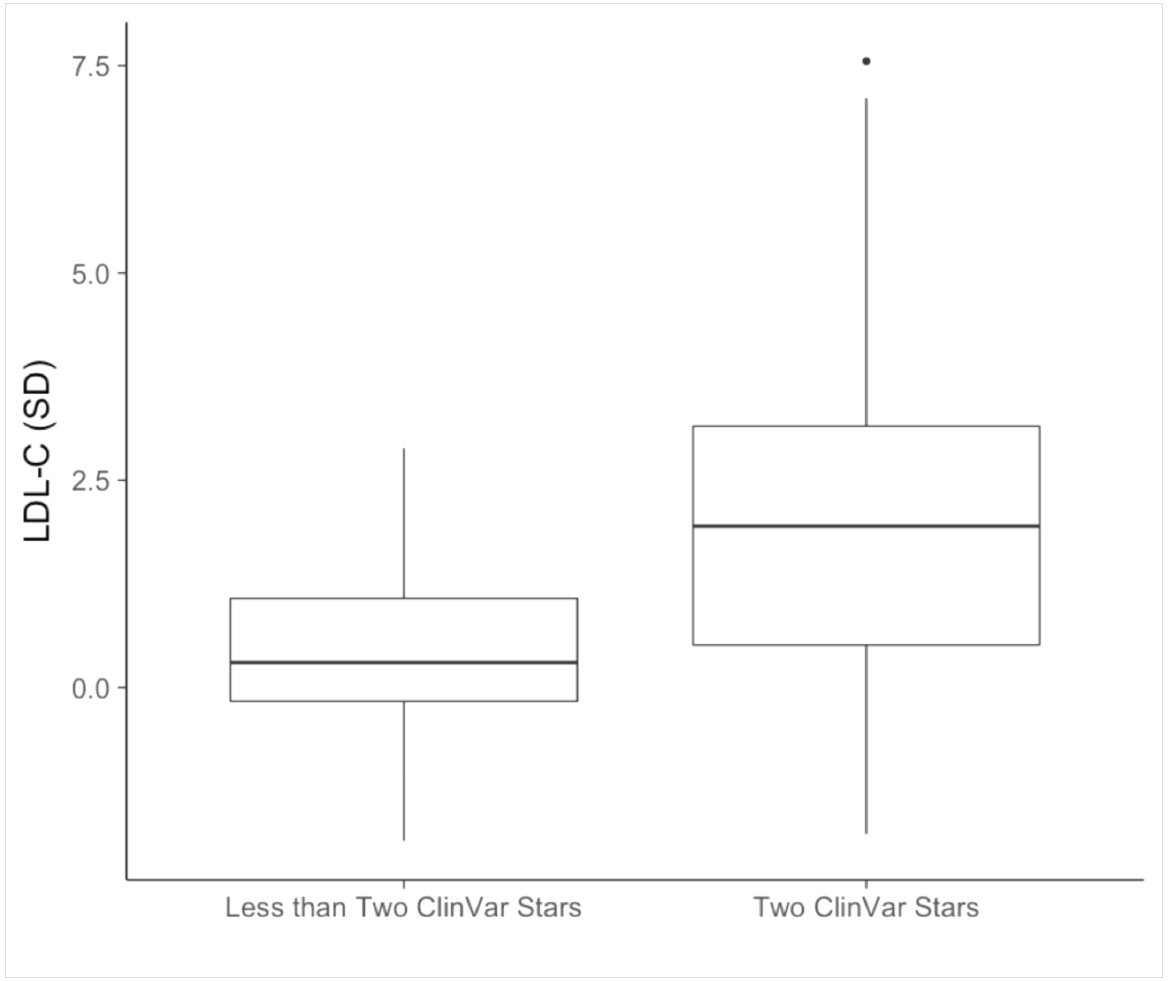

Supplementary Figure 5: Boxplot displaying the interquartile range of LDL-C in patient participants with P/LP *LDLR* missense variants with less than two stars (n=44) or two stars (n=90) in ClinVar. LDL-C was 1.66 SD (95% CI: 1.02, 2.31; $p = 1.24 \times 10^{-6}$) higher in individuals with two-star missense variants compared to individuals with one- or zero- star missense variants.

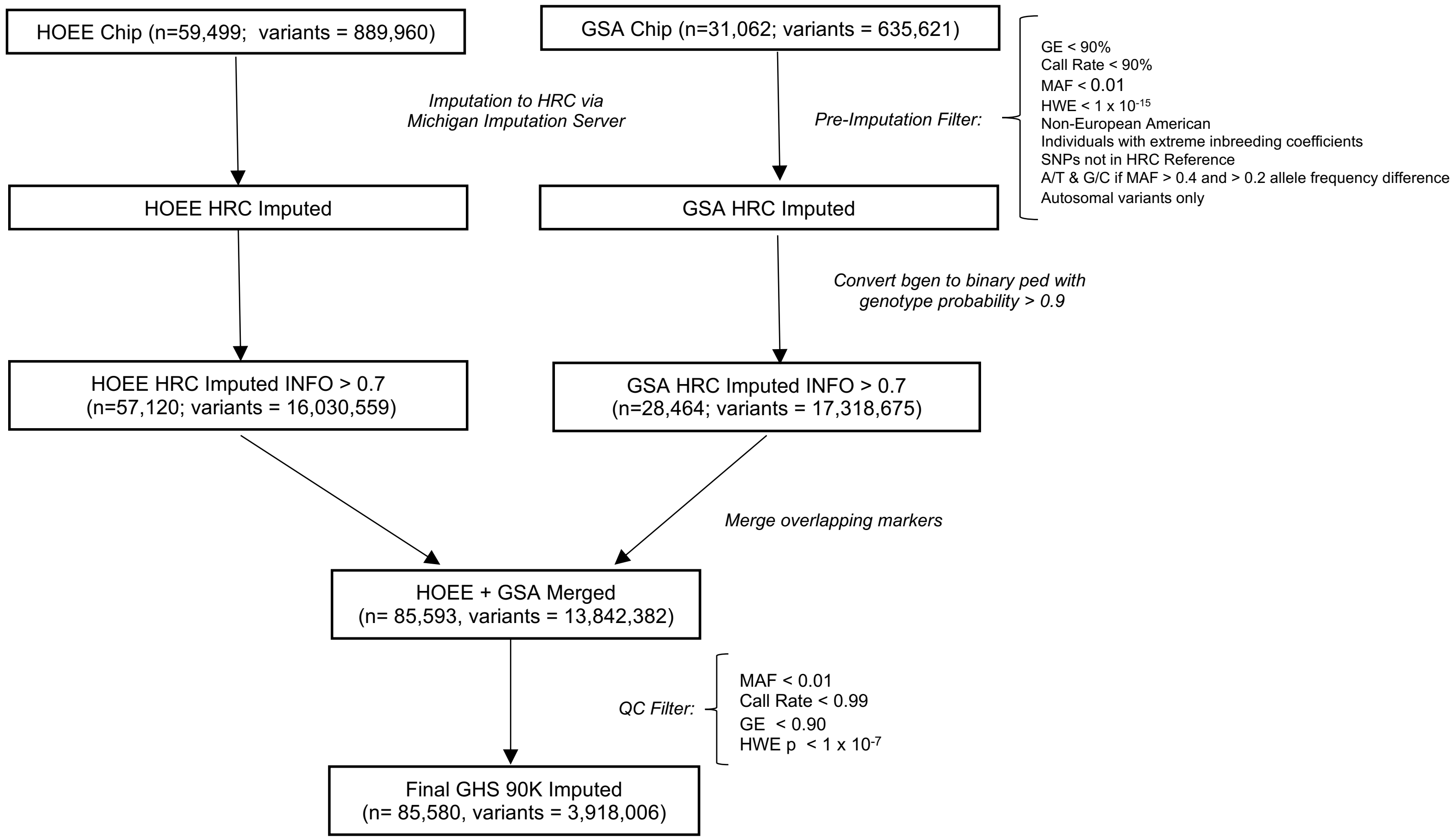

Supplementary Figure 6: Workflow describing imputation, QC, and merging of genotype data used in this study for polygenic scoring. Strict QC was applied to the final dataset to remove technical artifacts that may arise from merging the GSA and HOEE genotype platforms.

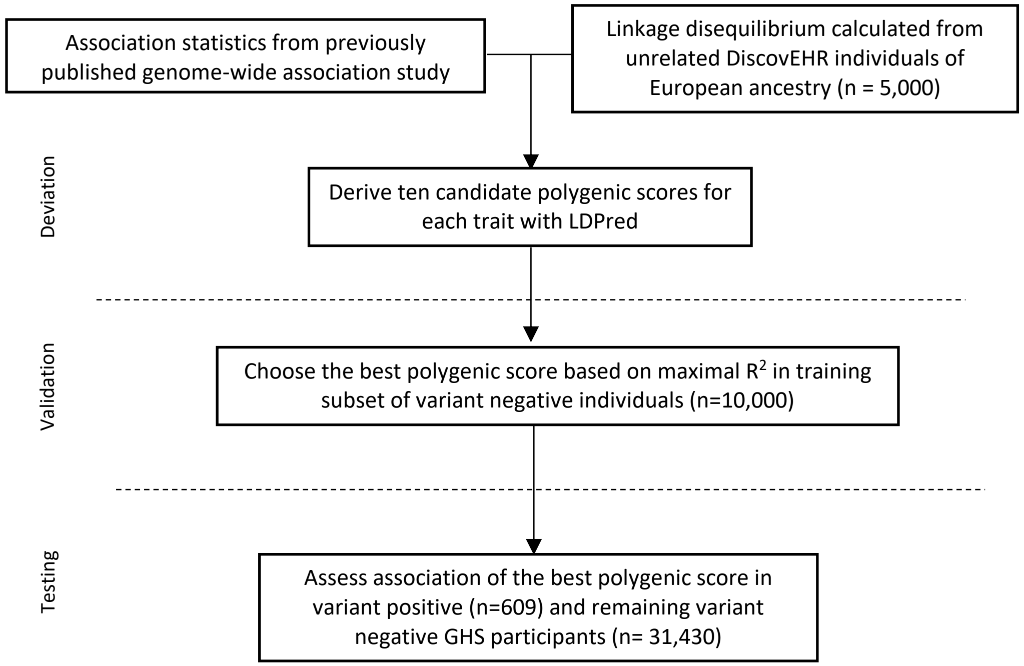

Supplementary Figure 7: Polygenic score workflow presented with the same design as Khera et al. (2018)[1].

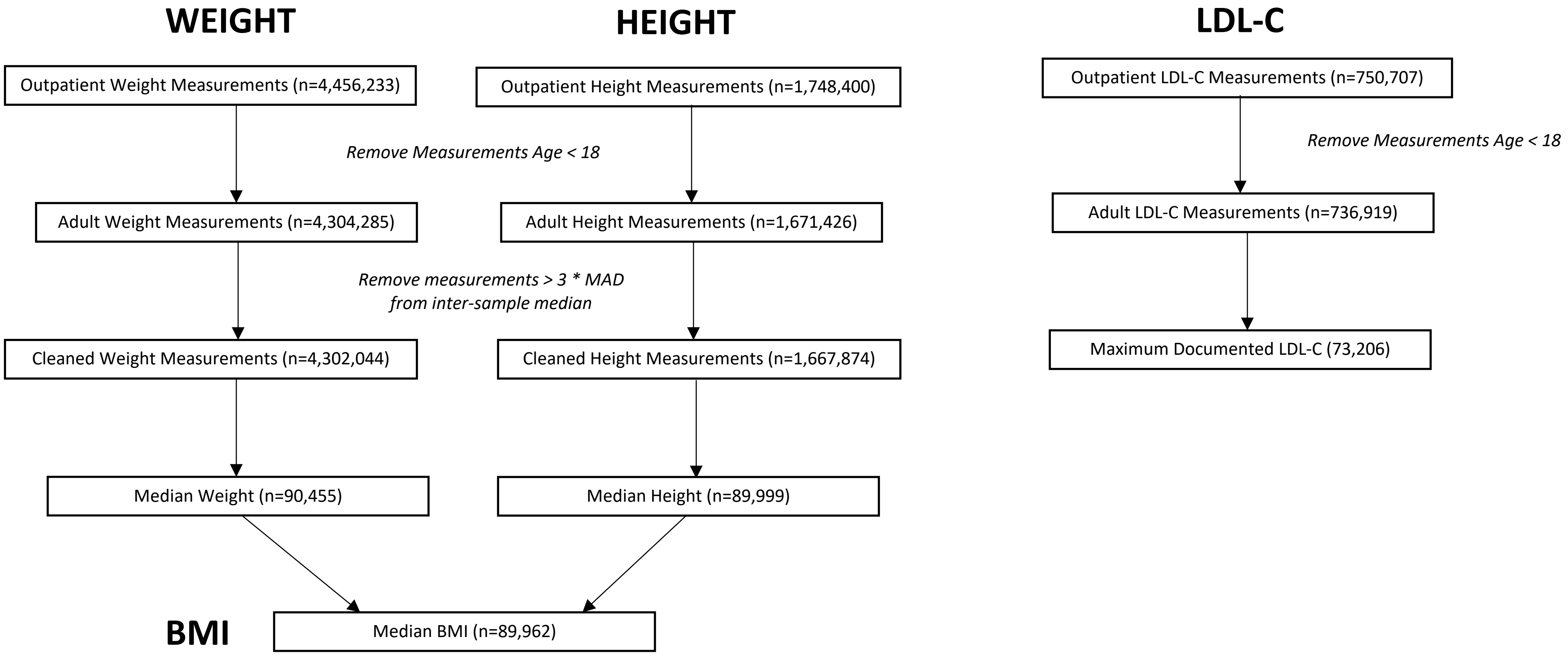

Supplementary Figure 8: Quality control and development of quantitative phenotypes derived from outpatient measurements. Height was recorded to the nearest inch, weight to the nearest pound, and LDL-C to the nearest mg/dL. Height and weight were converted to metric units. All phenotype values were residualized for Age, PC1-6, and genotype batch separately by sex in all available unrelated samples of European descent.

LDPred (ρ)	Variance Explained (R^2) Height	BMI	LDL-C
Infinite	**(0.217)**	0.106	0.023
1	0.195	**(0.109)**	0.024
0.3	0.193	0.101	0.036
0.1	0.162	0.069	0.561
0.03	0.142	0.042	**(0.079)**
0.01	0.087	0.029	0.024
0.003	0.070	0.019	0.015
0.001	0.039	0.005	0.017
0.0003	0.058	0.002	0.022
0.0001	0.049	0.003	0.029

Supplementary Table 1: Performance of LDPred polygenic scores in the validation cohort (n=10,000) at different increments of ρ, a prior to the LDPred model that accounts for the proportion of variants assumed to be causal. The maximal performing ρ for each phenotype is indicated with bold text and parentheses.

Supplementary Table 2: Test for equality between an extreme polygenic score (100th percentile) and RGD-causing variants

Trait	RGD	Extreme PGS	RGD Beta-Estimate* (95% CI)	P-Value (Uncorrected)	P-Value (Corrected)	RGD Sample Size	Extreme PGS Sample Size
Height	47,XXX	100th Percentile (Females)	-0.36 (-0.70, -0.02)	0.05	0.20	42	164
	47,XXY	100th Percentile (Males)	-0.72 (-1.06, -0.38)	4.41×10^{-5}	1.76×10^{-4}	44	151
	47,XYY	100th Percentile (Males)	0.04 (-0.39, 0.48)	0.84	1	24	151
	45,X	1st Percentile (Females)	-0.81 (-1.24, -0.37)	3.46×10^{-4}	1.38×10^{-3}	19	176
BMI	Melanocortin 4 Receptor Deficiency	100th Percentile	-0.32 (-0.67, 0.02)	0.06	0.18	58	315
	16p11.2 Deletion	100th Percentile	0.38 (-0.02, 0.77)	0.06	0.18	44	315
	16p11.2 Duplication	1st Percentile	0.23 (0.03, 0.43)	0.02	0.06	50	316
LDL-C	*LDLR* FH	100th Percentile	1.84 (1.53, 2.14)	1.60×10^{-28}	6.4×10^{-28}	146	315
	APOB FH	100th Percentile	0.76 (0.48, 1.04)	1.59×10^{-7}	6.36×10^{-7}	87	315
	PCSK9 FHBL	1st Percentile	0.06 (-0.19, 0.32)	0.61	1	42	315
	APOB FHBL	1st Percentile	-0.81 (-1.06, -0.56)	5.15×10^{-10}	2.06×10^{-9}	53	315

*A negative RGD beta-estimate indicates the effect size of the RGD is less than an extreme polygenic score

RGD: Rare Genetic Disorder
CI: Confidence Interval
FH: Familial Hypercholesterolemia
FHBL: Familial Hypobetalipoproteinemia

Supplementary Table 3: Spearman's Non-parametric test of the correlation between polygenic scores and quantitative phenotypes.

Trait	RGD	Spearman Rho	P-Value
Height	Variant Negative	0.45	$< 1 \times 10^{-300}$
	47,XXX	0.51	7.32×10^{-4}
	47,XXY	0.33	2.78×10^{-2}
	47, XYY	0.18	3.91×10^{-1}
	45,X	0.45	5.32×10^{-2}
BMI	Variant Negative	0.33	$< 1 \times 10^{-300}$
	Melanocortin 4 Receptor Deficiency	0.41	1.23×10^{-3}
	16p11.2 Deletion	0.16	3.12×10^{-1}
	16p11.2 Duplication	0.37	7.40×10^{-3}
LDL-C	Variant Negative	0.28	$< 1 \times 10^{-300}$
	LDLR FH	0.19	2.16×10^{-2}
	APOB FH	0.15	1.60×10^{-1}
	APOB FHLB	0.40	3.18×10^{-3}
	PCSK9 FHLB	0.45	3.04×10^{-3}

RGD: Rare Genetic Disorder
FH: Familial Hypercholesterolemia
FHBL: Familial Hypobetalipoproteinemia

Supplementary Table 4: Effect sizes of rare pathogenic variants adjusted for polygenic scores

Trait	RGD	RGD Beta (95% CI)	RGD P-Value	RGDAdjPGS Beta (95% CI)	RGDAdjPGS P-Value
Height	47,XXX	0.93 (0.64, 1.23)	1.46×10^{-9}	0.97 (0.70, 1.23)	1.56×10^{-12}
	47,XXY	0.56 (0.26, 0.85)	2.22×10^{-4}	0.55 (0.28, 0.81)	3.62×10^{-5}
	47,XYY	1.32 (0.92, 1.72)	8.67×10^{-11}	1.27 (0.91, 1.62)	2.32×10^{-12}
	45,X	-1.91 (-2.37, -1.47)	6.52×10^{-17}	-1.91 (-2.31, -1.52)	4.58×10^{-21}
BMI	Melanocortin 4 Receptor Deficiency	0.64 (0.39, 0.90)	9.03×10^{-7}	0.59 (0.36, 0.84)	1.32×10^{-06}
	16p11.2 Deletion	1.34 (1.05, 1.64)	4.80×10^{-19}	1.38 (1.10, 1.65)	1.14×10^{-22}
	16p11.2 Duplication	-0.52 (-0.80, -0.25)	2.09×10^{-4}	-0.63 (-0.89, -0.37)	2.48×10^{-06}
LDL-C	*LDLR* FH	2.49 (2.33, 2.65)	1.15×10^{-208}	2.47 (2.32, 2.63)	3.16×10^{-218}
	APOB FH	1.42 (1.21, 1.62)	7.39×10^{-42}	1.38 (1.18, 1.57)	2.57×10^{-43}
	PCSK9 FHBL	-0.72 (-1.01, -0.43)	1.55×10^{-6}	-0.78 (-1.06, -0.49)	6.41×10^{-8}
	APOB FHBL	-1.59 (-1.86, -1.33)	8.49×10^{-33}	-1.62 (-1.87 -1.37)	7.63×10^{-37}

RGD: Rare Genetic Disorder
RGDAdjPGS: RGD adjusted for Polygenic Score
CI: Confidence Interval
FH: Familial Hypercholesterolemia
FHBL: Familial Hypobetalipoproteinemia

Supplementary Table 5: Mean of standardized quantitative phenotypes across tertiles of the polygenic score by rare genetic disorders.

RGD	Tertile 1	Tertile 2	Tertile 3
47, XXX	0.39 ± 0.26	0.91 ± 0.24	1.83 ± 0.31
47, XXY	0.11 ± 0.24	0.51 ± 0.21	1.05 ± 0.36
47, XYY	0.68 ± 0.40	1.67 ± 0.45	1.48 ± 0.35
45,X	-2.65 ± 0.25	-2.63 ± 0.30	-0.35 ± 0.68
Melanocortin 4 Receptor Deficiency	-0.02 ± 0.29	0.74 ± 0.32	1.00 ± 0.18
16p11.2 Deletion	1.06 ± 0.32	1.44 ± 0.35	1.56 ± 0.35
16p11.2 Duplication	-0.89 ± 0.25	-0.71 ± 0.14	-0.18 ± 0.17
LDLR FH	1.88 ± 0.26	2.58 ± 0.30	2.93 ± 0.35
APOB FH	1.35 ± 0.24	1.24 ± 0.25	1.59 ± 0.23
APOB FHLB	-2.18 ± 0.14	-1.43 ± 0.27	-1.26 ± 0.30
PCSK9 FHLB	-1.22 ± 0.17	-1.74 ± 0.25	-0.42 ± 0.17

RGD: Rare Genetic Disorder
FH: Familial Hypercholesterolemia
FHBL: Familial Hypobetalipoproteinemia
Standard error of the mean is included after the ± symbol. A value of 0 indicates the phenotype is approximately equal to the mean of the variant negative population.

Supplementary Table 6: Tests for equality of PGS beta-estimates in RGD+ and RGD- individuals

Trait	Rare Genetic Disorder	Test Statistic	P-Value (Uncorrected)	P-Value (Corrected)
Height	47,XXX	-0.40	0.69	1
	47,XXY	-0.05	0.96	1
	47,XYY	0.78	0.43	1
	45,X	-1.40	0.16	0.65
BMI	Melanocortin 4 Receptor Deficiency	-1.37	0.17	0.68
	16p11.2 BP4-5 Deletion	-0.16	0.87	1
	16p11.2 BP4-5 Duplication	0.14	0.89	1
LDL-C	*LDLR* FH	-1.25	0.21	0.84
	APOB FH	0.81	0.42	1
	APOB FHLB	-0.16	0.88	1
	PCSK9 FHLB	-0.63	0.53	1

RGD: Rare Genetic Disorder
CI: Confidence Interval
FH: Familial Hypercholesterolemia
FHBL: Familial Hypobetalipoproteinemia

Supplementary Table 7: Median LogR thresholds for calling sex chromosomal aneuploidy in DiscovEHR on the HOEE and GSA platforms

Aneuploidy	GSA mLRR Threshold	HOEE mLRR Threshold	EHR-Documented Sex
47,XXX	> 0.09	> 0.15	Female
47,XXY	> 0.1	> -0.01	Male
47,XYY	> 0.10	> -0.2	Male
45,X and 45,X/46,XX	< -0.28	< -0.20	Female

GSA - Global Screening Array
HOEE - Human Omni Exome Express
mLRR - Median Log R Ratio

Supplementary Table 8: Prevalence of rare genetic disorders in DiscovEHR.

Rare Genetic Disorder	Samples Identified	Variant Negative	Prevalence in DiscovEHR (%)	Included in Study	Sample Inclusion Criteria for Prevalence
47,XXX	46	48,427	0.095	42	EHR-Documented Females Passing Array Intensity QC
47,XXY	47	31,834	0.148	44	EHR-Documented Males Passing Array Intensity QC
47,XYY	27	31,834	0.085	24	EHR-Documented Males Passing Array Intensity QC
45,X	21	48,427	0.043	19	EHR-Documented Females Passing Array Intensity QC
Melanocortin 4 Receptor Deficiency	81	92,455	0.088	58	Passing WES QC
16p11.2 BP4-5 Deletion	58	90,620	0.064	44	Passing CLAMMS QC
16p11.2 BP4-5 Duplication	63	90,620	0.070	50	Passing CLAMMS QC
LDLR FH	233	92,455	0.252	146	Passing WES QC
APOB FH	127	92,455	0.137	87	Passing WES QC
PCSK9 FHBL	83	92,455	0.090	42	Passing WES QC
APOB FHBL	85	92,455	0.092	53	Passing WES QC

Supplementary Note 1: Comparisons of Variance Explained by PGSs in DiscovEHR with Other Cohorts

In the testing cohort, the variance explained by the PGS_{HEIGHT} (21.2%) and PGS_{BMI} (11.5%) were similar to those reported in the combined GWAS meta-analysis publication that produced the summary statistics. In the Health and Retirement Study (HRS) using associated SNPs (p<0.001) the variance explained by the PGS_{HEIGHT} and PGS_{BMI} scores were reported to be ~24.4% and ~8.6%, respectively. While we observe an improvement in the PGS_{BMI}, we note that height in the DiscovEHR data is measured and recorded to the nearest inch, which may reduce the variance explained by the PGS_{HEIGHT} relative to cohorts that record heights to the nearest centimeter (UK Biobank) or quarter-inch (HRS).

Our PGS_{LDL-C} score is more predictive than a recent PGS analysis in the Million Veteran Program (MVP),] which constructed a PGS of genome-wide significant SNPs (n=223) from summary statistics of an exome-array based association study[2,3]. This study reported that the variance explained was 4.1% when using maximum documented LDL-C as the phenotype. On the other hand, an analysis of a PGS_{LDL-C} by the NIH/NHLBI Trans-Omics for Precision Medicine (TOPMed) research program on 16,324 individuals with whole-genome sequence (WGS) data reported the effect size of a high PGS_{LDL-C} (top 5% of distribution) to be approximately 33.07 mg/dL in European Americans. Relative to the TOPMed analysis, we report a smaller effect size of a high PGS_{LDL-C} using the same percentile at 23.57 mg/dL.

Supplementary Note 2: Non-Parametric Analysis of PGS and Variable Expressivity

The non-parametric Spearman's rank-order correlation yielded similar results as compared with linear regression, with the exceptions of 45,X and 47,XXY, which trended toward and met nominal significance, respectively. The Spearman's correlation coefficients (ρ) of the PGS and

trait-expression in these two RGDs were similar to that of the general population (Supplemental Table 3).

Supplementary Note 3: Members of the Geisinger-Regeneron DiscovEHR Collaboration

Regeneron Genetics Center

Goncalo Abecasis, Ph.D., Aris Baras, M.D., Michael Cantor, M.D., Giovanni Coppola, M.D., Aris Economides, Ph.D., Luca Lotta, M.D., Ph.D., John D. Overton, Ph.D., Jeffrey G. Reid, Ph.D., Alan Shuldiner, M.D, Christina Beechert, Caitlin Forsythe, M.S., Erin D. Fuller, Zhenhua Gu, M.S., Michael Lattari, Alexander Lopez, M.S., John D. Overton, Ph.D., Thomas D. Schleicher, M.S., Maria Sotiropoulos Padilla, M.S., Karina Toledo, Louis Widom, Sarah E. Wolf, M.S., Manasi Pradhan, M.S., Kia Manoochehri, Ricardo H. Ulloa, Xiaodong Bai, Ph.D., Suganthi Balasubramanian, Ph.D., Leland Barnard, Ph.D., Andrew Blumenfeld, Gisu Eom, Lukas Habegger, Ph.D., Young Hahn, Alicia Hawes, B.S., Shareef Khalid, Jeffrey G. Reid, Ph.D., Evan K. Maxwell, Ph.D., William Salerno, Ph.D., Jeffrey C. Staples, Ph.D., Ashish Yadav, M.S., Marcus B. Jones, Ph.D., and Lyndon J. Mitnaul, Ph.D.

Geisinger

W. Andrew Faucett, Christopher Still, F. Daniel Davis, David J. Carey, Derek Boris, Dustin N. Hartzel, Joseph B. Leader, Lance J. Adams, H. Lester Kirchner, Matthew T. OetjensMarc Williams, J. Neil Manus, Raghu P. Metpally, Ryan D. Colonie, Sarah A. Pendergrass, Tooraj Mirshahi, Jen Wagner, Huntington F. Willard, Christa L. Martin, and David H, Ledbetter Ph.D., and Thomas Nate Person

References

1. Khera, A. V. *et al.* Genome-wide polygenic scores for common diseases identify individuals with risk equivalent to monogenic mutations. *Nat. Genet.* 1 (2018).
2. Klarin, D. *et al.* Genetics of blood lipids among ~300,000 multi-ethnic participants of the Million Veteran Program. *Nat. Genet.* **50**, 1514–1523 (2018).
3. Liu, D. J. *et al.* Exome-wide association study of plasma lipids in >300,000 individuals. *Nat. Genet.* **49**, 1758–1766 (2017).
