# Supplementary material for: Quantifying the polygenic contribution to variable expressivity in eleven rare genetic disorders
Source: Nat Commun. 2019 Oct 25;10:4897. doi: 10.1038/s41467-019-12869-0 (PMC6814771; doi:10.1038/s41467-019-12869-0)
Supplement: Supplementary file 4 — Reporting Summary [file 41467_2019_12869_MOESM4_ESM.pdf]

nature research

Corresponding author(s): David H. Ledbetter

Last updated by author(s): 03-21-2019

Reporting Summary

Nature Research wishes to improve the reproducibility of the work that we publish. This form provides structure for consistency and transparency in reporting. For further information on Nature Research policies, see Authors & Referees and the Editorial Policy Checklist .

Statistics

For all statistical analyses, confirm that the following items are present in the figure legend, table legend, main text, or Methods section.

n/a	Confirmed	
[ ]	[x]	The exact sample size (*n*) for each experimental group/condition, given as a discrete number and unit of measurement
[x]	[ ]	A statement on whether measurements were taken from distinct samples or whether the same sample was measured repeatedly
[ ]	[x]	The statistical test(s) used AND whether they are one- or two-sided<br>*Only common tests should be described solely by name; describe more complex techniques in the Methods section.*
[ ]	[x]	A description of all covariates tested
[ ]	[x]	A description of any assumptions or corrections, such as tests of normality and adjustment for multiple comparisons
[ ]	[x]	A full description of the statistical parameters including central tendency (e.g. means) or other basic estimates (e.g. regression coefficient) AND variation (e.g. standard deviation) or associated estimates of uncertainty (e.g. confidence intervals)
[ ]	[x]	For null hypothesis testing, the test statistic (e.g. *F*, *t*, *r*) with confidence intervals, effect sizes, degrees of freedom and *P* value noted<br>*Give P values as exact values whenever suitable.*
[ ]	[x]	For Bayesian analysis, information on the choice of priors and Markov chain Monte Carlo settings
[x]	[ ]	For hierarchical and complex designs, identification of the appropriate level for tests and full reporting of outcomes
[ ]	[x]	Estimates of effect sizes (e.g. Cohen's *d*, Pearson's *r*), indicating how they were calculated

Our web collection on statistics for biologists contains articles on many of the points above.

Software and code

Policy information about availability of computer code

Data collection	Plink v1.9 - open source<br>LDPred v0.6 - open source<br>PRIMUS (Pedigree Reconstruction and Identification of a Maximum Unrelated Set) - open source<br>VEP (Variant Effect Predictor) - open source
Data analysis	Statistical analyses were conducted using R version 3.4.3 software (The R Foundation)

For manuscripts utilizing custom algorithms or software that are central to the research but not yet described in published literature, software must be made available to editors/reviewers. We strongly encourage code deposition in a community repository (e.g. GitHub). See the Nature Research guidelines for submitting code & software for further information.

Data

Policy information about availability of data

All manuscripts must include a data availability statement. This statement should provide the following information, where applicable:

- Accession codes, unique identifiers, or web links for publicly available datasets
- A list of figures that have associated raw data
- A description of any restrictions on data availability

Polygenic scores in this manuscript were derived from publicly available summary statistics from genome-wide association studies performed by the GIANT and GLGC consortium. All RGD-causing variants reported and used for the analyses in this study are listed in the methods and supplement.

Field-specific reporting

Please select the one below that is the best fit for your research. If you are not sure, read the appropriate sections before making your selection.

☒ Life sciences ☐ Behavioural & social sciences ☐ Ecological, evolutionary & environmental sciences

For a reference copy of the document with all sections, see nature.com/documents/nr-reporting-summary-flat.pdf

Life sciences study design

All studies must disclose on these points even when the disclosure is negative.

Sample size	Sample size was based on the 92,455 patient-participants sequenced as part of the DiscovEHR study. An unrelated subset of European-American samples with variant-data passing QC threshold were included in this study. Sample sizes of variant-positive individuals were limited by the frequency of disease-causing variants in DiscovEHR.
Data exclusions	We limited out study to samples of European ancestry. We excluded one individual from each 1st, 2nd, and 3rd degree (if confirmed by PRIMUS) pair. We limited our study to patient-participants with EHR data of the affected phenotype. Sample were excluded if they failed pre-defined exome-sequencing or array genotyping QC metrics including call rates, high imbreeding coefficients, or discordant sex. A full description of our sample inclusion criteria is described in the methods and supplement.
Replication	All polygenic scores replicated across validation and testing cohorts in variant negative individuals.
Randomization	Variant-negative individuals were randomized into either a validation cohort for optimizing PGS scores or a testing cohort for their application.
Blinding	Polygenic score calculation and variant curation were performed by investigators blinded to phenotypic status.

Reporting for specific materials, systems and methods

We require information from authors about some types of materials, experimental systems and methods used in many studies. Here, indicate whether each material, system or method listed is relevant to your study. If you are not sure if a list item applies to your research, read the appropriate section before selecting a response.

Materials & experimental systems

n/a	Involved in the study	
☒	☐	Antibodies
☒	☐	Eukaryotic cell lines
☒	☐	Palaeontology
☒	☐	Animals and other organisms
☐	☒	Human research participants
☒	☐	Clinical data

Methods

n/a	Involved in the study	
☒	☐	ChIP-seq
☒	☐	Flow cytometry
☒	☐	MRI-based neuroimaging

Human research participants

Policy information about studies involving human research participants

Population characteristics	In the DiscovEHR testing cohort (average age at last visit = 60.42 years; 57.7% female), the mean (SD) of the three quantitative phenotypes were LDL-C: 137.67 mg/dL (41.63); female height 161.94 cm (6.66); male height: 176.69 cm (7.20); and BMI 31.94 kg/m2 (7.72)
Recruitment	Individuals were recruited from Geisinger, a health care system of northeastern and central Pennsylvania. This population is primarly of European descent and over samples patients in the age range of 60-89 and under samples adults younger than 30. Recruitment does not depend on a particular condition or diagnosis and participants have been enrolled from a large number of diverse clinics.
Ethics oversight	Geisinger Institutional Review Board

Note that full information on the approval of the study protocol must also be provided in the manuscript.
