# Supplementary material for: Quantifying the polygenic contribution to variable expressivity in eleven rare genetic disorders
Source: Nat Commun. 2019 Oct 25;10:4897. doi: 10.1038/s41467-019-12869-0 (PMC6814771; doi:10.1038/s41467-019-12869-0)
Supplement: Supplementary file 5 — Description of Additional Supplementary Files [file 41467_2019_12869_MOESM5_ESM.pdf]

Title: Supplemental Data 1

Description: List of RGD-causing SNV and indels included in this study
